# Privacy preserving identification of population stratification for collaborative genomic research

**DOI:** 10.1093/bioinformatics/btad274

**Published:** 2023-06-30

**Authors:** Leonard Dervishi, Wenbiao Li, Anisa Halimi, Xiaoqian Jiang, Jaideep Vaidya, Erman Ayday

**Affiliations:** Computer and Data Sciences, Case Western Reserve University, OH 44106, United States; Computer and Data Sciences, Case Western Reserve University, OH 44106, United States; Research, IBM, D15HN66, Ireland; School of Biomedical Informatics, University of Texas Health Science Center at Houston, TX 77030, United States; Management Science and Information Systems Department, Rutgers University, NJ 07102, USA; Computer and Data Sciences, Case Western Reserve University, OH 44106, United States

## Abstract

The rapid improvements in genomic sequencing technology have led to the proliferation of locally collected genomic datasets. Given the sensitivity of genomic data, it is crucial to conduct collaborative studies while preserving the privacy of the individuals. However, before starting any collaborative research effort, the quality of the data needs to be assessed. One of the essential steps of the quality control process is population stratification: identifying the presence of genetic difference in individuals due to subpopulations. One of the common methods used to group genomes of individuals based on ancestry is principal component analysis (PCA). In this article, we propose a privacy-preserving framework which utilizes PCA to assign individuals to populations across multiple collaborators as part of the population stratification step. In our proposed client-server-based scheme, we initially let the server train a global PCA model on a publicly available genomic dataset which contains individuals from multiple populations. The global PCA model is later used to reduce the dimensionality of the local data by each collaborator (client). After adding noise to achieve local differential privacy (LDP), the collaborators send metadata (in the form of their local PCA outputs) about their research datasets to the server, which then aligns the local PCA results to identify the genetic differences among collaborators’ datasets. Our results on real genomic data show that the proposed framework can perform population stratification analysis with high accuracy while preserving the privacy of the research participants.

## 1 Introduction

Collaborative studies that pool large data from multiple sources are generally more powerful statistics, and as a result, are beneficial to all the participating parties. To ensure accurate outcomes of collaborative studies, the quality and consistency of the data among the datasets of the collaborators need to be ensured (Turner et al. 2011; Zuvich et al. 2011). A trivial solution is to pool the dataset of each party at a centralized environment and analyze the data. However, the dataset of each party might not always be shared, which could lead to sensitive information about the research participants being revealed.

There are known techniques to preserve the privacy for genomic data processing such as meta-analysis, cryptographic solutions, and differential privacy (DP)-based solutions. In meta-analysis, collaborators exchange aggregate statistics in order to obtain global statistics of a specific study. In cryptographic solutions, the collaborators are able to perform the collaborative analysis over the encrypted data. DP-based solutions allow the collaborators to exchange perturbed data or statistics between them under some privacy guarantees. Each of these solutions comes with their drawbacks, as discussed below.

- Sharing aggregate statistics as part of meta-analysis still poses privacy risk for the dataset participants (Zerhouni and Nabel 2008). Although the aggregate statistics are anonymized, it is possible to re-identify individuals based on the information that is shared. This could lead to potential harm if the data are used for malicious purposes. It is important to be careful when sharing aggregate statistics and to make sure that only information that cannot be used to identify individuals is shared.
- Cryptographic techniques are often used to provide high privacy guarantees for datasets. However, these techniques are not applicable for large-scale datasets. This is because the computational resources required to implement cryptographic techniques on large datasets are prohibitive (Yakoubov et al. 2014).
- DP-based solutions add a large amount of noise to the data they are protecting. This makes it difficult for an attacker to learn anything about the underlying data from the noise. However, it also makes it difficult for legitimate users of the data to get accurate results from queries (Sarathy and Muralidhar 2011).

Due to aforementioned limitation, existing solutions either do not scale up or may not be able to provide accurate information about the genetic makeup of a population. As a result, these technologies may not be able to provide ideal solutions to our problems in collaborative genomic research.

One of the main quality control steps that need to be considered in collaborative studies is population stratification (i.e. population structure). This can occur when the genetic difference in case-control groups occurs due to differences in ancestry, rather than the association to the phenotype (trait) of interest. Previous genetic studies (Patterson et al. 2006; Price et al. 2006) have been widely using principal component analysis (PCA), a popular technique which analyzes and identifies patterns within a data, for population stratification analysis. To prevent bias caused by population stratification, a possible approach is to make sure that the study participants are selected from a relatively homogenous population (Turner et al. 2011). Therefore, in this work, we focus on performing population stratification analysis (i.e. assigning individuals to populations) in collaborative studies in a privacy-preserving manner by using PCA.

We consider an environment in which multiple collaborators (researchers) are present whose aim is to determine whether the individuals that are present in their local datasets belong to multiple populations and a centralized entity (server) which is used to capture the population structure and assign the population cluster to each data point (individual). There exist a few major population clusters that are widely accepted by the scientific community and most of the individuals fall into one of those clusters, but due to genetic admixture, which occurs when individuals from different populations interbreed, there are individuals which have mixed ancestry. A detailed discussion is provided in Supplementary Material. In our approach, we classify such individuals to the closest population cluster. Our main goals are to obtain an accurate PCA outcome of the federated dataset of all researchers and to preserve the privacy of the samples (data contributors) in researchers’ datasets. The challenge lies in the fact that due to privacy concerns, researchers cannot send their local datasets to the server (or to each other) for a combined PCA. One trivial way to perform combined PCA across different researchers is to let each researcher conduct PCA on their local datasets, and then send the PCA results to the server. However, combining independent PCA results at the server to identify the relative distance between the data points is not possible. Individual PCA results are generated by linear combinations of the local variables and since they are dependent on the distinct local dataset, they cannot be combined.

Thus, we propose a privacy-preserving collaborative PCA scheme between researchers and the server. The server initially trains a PCA model using a publicly available genomic dataset which contains individuals of various populations. Next, the trained PCA model is sent to the researchers and the researchers conduct their local PCA using this model. As a result, each researcher obtains the projection of individuals in their datasets with respect to the trained PCA model. To achieve LDP (ϵ-LDP, where ϵ is the privacy parameter), and hence to protect the privacy of the records in their local datasets, researchers add Laplacian noise (i.e. noise based on Laplacian distribution) to each sample and send some metadata, which contains the PCA data points (principal components) and the hashed IDs of each sample, to the server. The server then calculates the coordinates for all the users coming from multiple researchers, combines the PCA results coming from multiple researchers, and identifies the population substructure. Finally, it sends back to each researcher the hashed user IDs and the label of the population cluster they belong to.

We quantify the privacy risk of sharing such metadata. We consider membership inference risk as the main vulnerability and we show that the privacy risk introduced by our framework at the server is below the baseline risk of sharing summary statistics as part of genetic studies, which is allowed by a lot of institutions, including NIH (NIH management of genomic summary results access 2018). We evaluate our framework in terms of both utility and privacy. From the obtained results, we observe that the accuracy of correctly identifying population clusters increases when the server trains the PCA model on a public genomic dataset consisting of various populations. We also monitor the effect of the number of populations that are present in the researchers’ datasets for various privacy parameter values. Our framework achieves a precision of 0.89 and a recall of 0.88 when the researchers’ datasets contain individuals from multiple populations. For the same setup, it achieves a membership inference power of 0.19 (which is a significantly low value indicating high privacy). The proposed framework achieves a higher power (a power of 0.39) when the researchers’ datasets contain only one population, which is still lower than the power due to sharing the genome-wide association studies (GWAS) statistics (the baseline risk).

## 2 Related work

*PCA and population stratification*: PCA is a popular technique used to analyze and identify the patterns and relationships within the data. PCA is used in image compression (Gaidhane et al. 2010), facial recognition (Gottumukkal and Asari 2004), medical data correlation (Qureshi et al. 2017), and quantitative finance (Yu et al. 2014). PCA can also be used to detect population substructures in genetic data. Population stratification is one of the crucial steps in quality control procedures performed before GWAS in order to guarantee the homogeneity of the data. PCA can also identify continuous population structure in various association studies (Novembre and Stephens 2008). Paschou et al. (2007) introduce a novel approach that uses PCA-correlated genetic markers (a smaller proportion and more significant set of markers) to reproduce the population substructure and evaluate it against the PCA findings on the whole dataset. Lee et al. (2009) initially apply PCA to the genotype data of a population, select the most significant principal components by using Tracy-Widom distribution, and finally use generic clustering algorithms to determine the population substructure. Note that none of the aforementioned studies takes in consideration the privacy of the dataset participants (especially, in collaborative studies), which is one of the main goals of our work. Cho et al. (2018) propose a feasible secure multiparty computation (MPC) protocol in order to perform secure genome-wide association analysis. As part of their protocol, they also perform quality control and population stratification correction in the local GWAS setting. However, in our work, we are interested in performing the population stratification analysis in a federated way (considering samples across multiple collaborators).

*Privacy-preserving PCA*: When computation requires more power than is available locally, outsourcing to the cloud may be necessary. However, in some cases the data cannot be sent to the cloud due to privacy concerns. Zhang et al. (2020) propose a secure outsourcing protocol which uses eigen decomposition and singular value decomposition (SVD) techniques. They demonstrate the applicability of the proposed protocol for face recognition tasks. However, it is not applicable to the scenario when the data are owned by multiple entities (which is the case we consider). Ostrak et al. (2021) redesign the stratification algorithms such as EIGENSTRAT (Price et al. 2006) using secure MPC and trusted execution environment. Their proposed approach guarantees data privacy, but it is computationally expensive [e.g. the runtime is >7 h for 2000 single-nucleotide polymorphisms (SNPs) and 868 samples].

*Collaborative PCA*: Researchers have also paid attention to using PCA in a collaborative manner. Kung et al. (2017) propose a privacy-preserving technique in which the data owners compress the data via collaborative learning so that the compressed data can be utilized only in the intended way, while preserving the privacy of the research participants. As part of the collaborative learning, each data owner needs to supply feature vectors to the cloud (server) and they use dimensionality reduction to compress the vectors with the most prominent tool being PCA for unsupervised learning and discriminant component analysis for supervised learning. Different from our work, they use PCA as a tool for compressive privacy by sending perturbed feature vectors to build the collaborative model (e.g. train the classification model) at the cloud, while we send dimensionality reduced data to be classified by the cloud model, which was trained beforehand. Their approach is not applicable to our scenario since the feature vectors generated by each data owner can only be used to train a classifier (such as for face or speech data), but they cannot be aggregated to determine the relative distances between individuals from different populations, which is a key requirement for our scenario.

## 3 Background information

In this section, we provide some background information about genomic data, LDP, and PCA.

### 3.1 Genomic background

The human genome consists of the complete set of DNA which is the hereditary material in humans. DNA comprises of four different building blocks called nucleotides (A, G, C, T). Human genome has 23 pairs of chromosomes, >20 000 genes, and 3 billion pair bases. The DNA sequence is the blueprint of developing from a cell to an organism and the encoded genes within DNA sequence are accountable for different traits in different people. An allele is a DNA sequence version found at a particular chromosomal location which typically consists of a single base (may also consist of a segment of bases). Most of the genome is identical between any two individuals, with only a small amount varying between people. This is also known as genetic variation, and the most frequent type of variation is called SNP. SNPs can be used as biological markers to identify genes that are associated with a certain disease. Most SNPs are biallelic, meaning that there exist two different nucleotides at a particular position. The most common allele is called the major allele, and the less common is known as the minor allele. In our work, we represent the value of a SNP as 0, 1, or 2 depending on the number of minor alleles it contains.

### 3.2 Local differential privacy

LDP (Duchi et al. 2013; Kairouz et al. 2014) is a variant of DP (Dwork, 2008), with a distributed architecture and provides strong guarantees for each individual’s privacy. DP incorporates a centralized trusted party that has access to the raw data. In contrast, LDP uses each user’s local dataset to perturb it and then sends it to a data collector. By definition, an algorithm *A* satisfies ϵ-LDP, if for any two user’s private data points $a_{1}$ & $a_{2}$ and output *b*: where ϵ is the privacy parameter. One way to achieve ϵ-LDP is to add noise to each data point. The main challenge is to determine the amount of noise to add to achieve LDP, while still maintaining a good level of utility. Several different mechanisms have been developed in the DP field to solve this problem, the most well-known of which is the Laplacian mechanism (Dwork et al. 2006). For any numerical function f(x):R→R, F(X) satisfies ϵ-LDP if we add Laplacian noise as follows: where *s* is the sensitivity of the function *f*. Sensitivity captures the maximum amount of change that a single data point can cause in the worst case to the output of the function f. In our work, we use the $l_{1}$ sensitivity to add Laplacian noise (Dwork and Roth 2013). By definition, $s=\underset{x,x^{\prime }\in R}{\max}||f(x)-f(x^{\prime })||$.


(1) $$Pr[A(a_{1})=b]\leq e^{\epsilon }Pr[A(a_{2})=b],$$

(2) $$F(x)=f(x)+\mathrm{Lap}\left(\frac{s}{\epsilon }\right),$$

Lap(λ) , where $\lambda =\frac{s}{\epsilon }$, denotes sampling from a Laplace distribution with scale λ and with a probability density function: where μ is a location parameter (μ=0 to have a symmetric distribution).


(3) $$\mathrm{pdf}(x|\mu ,\lambda )=\frac{1}{2\lambda }\text{exp}\;\left(-\frac{\left|x-\mu \right|}{\lambda }\right),$$

### 3.3 PCA and PCA training

The goal of PCA is to extract the largest sources of variation from high dimensional and inter-correlated datasets by computing new orthogonal variables called principal components, which are linear combinations of the original variables. PCA computes the eigenvectors and eigenvalues from the covariance matrix and sorts the eigenvectors according to the amount of explained variance (i.e. according to their corresponding eigenvalues in descending order). Generally, only the top eigenvectors are used to transform the data, and they are called principal components. The number of principal components that are obtained from PCA is adjustable.

Suppose a matrix *Z* has *I* observations (records), *J* variables, and rank *L* where *L* is the minimum number of variables that describes *I* observations (or the minimum number of observations that can be described by *J* variables). To train a PCA model on *Z*, and as a result, compute its components, SVD is used with $Z=P\Delta Q^{T}$, where *P* is the I×L matrix of left singular vectors, *Q* is the J×L matrix of right singular vectors, and Δ is the diagonal matrix of singular values (Abdi and Williams 2010). The new data points of any observation converted by the fitted principal components (i.e. trained PCA model) are called factor scores, denoted as *F*. *F* is the I×L matrix obtained as F=PΔ, or $F=P\Delta Q^{T}Q=ZQ$ since $Z=P\Delta Q^{T}$. The matrix *Q* (defined as components_ in scikit-learn; Pedregosa et al. 2011) is a projection matrix that transforms not only the observations in *Z* but also new or supplementary observations $Z_{\sup}$ (that are not in *Z*) into factor scores. The factor scores *F* for supplementary observations are obtained as $F_{\sup}^{T}=Z_{\sup}^{T}Q$. Thus, the PCA model $M^{s}$ (one of the main components of the proposed framework) consists of the number of principal components and the matrix *Q*.

## 4 System and threat models

In this section, we describe the system model together with the threat model.

### 4.1 System model

There are two types of parties involved in the identification of population substructure via PCA: (i) two or more researchers and (ii) a server. To identify population substructure across multiple researchers’ datasets, each researcher provides some metadata to the server. This metadata will be discussed in the next section. The server is able to identify all the populations and label to which population each individual belongs. Based on the received metadata, the server determines the population cluster of each individual in the federated dataset of all researchers. In the rest of the article, for the sake of simplicity, we only consider two researchers. However, our approach can be extended to multiple researchers. Depending on the study the researchers are conducting, they may decide to keep only the individuals that belong to the largest population in their combined dataset or the ones that belong to the smallest population. Note that such steps are not in the scope of our work, as we mainly focus on identifying population substructure across multiple researchers’ datasets.

### 4.2 Threat model

We consider honest researchers that have legitimate genomic datasets and who follow the protocol. On the other hand, we consider an honest-but-curious server. In other words, the server correctly conducts the computations and procedures, but it may try to infer sensitive information (about the individuals in researchers’ datasets) from the metadata sent by the researchers. The most well-known attacks to the genomic datasets include membership inference (Homer et al. 2008; Wang et al. 2009), attribute inference (Humbert et al. 2013), and de-anonymization attacks (Gymrek et al. 2013). In the aforementioned attacks, the adversary tries to exploit the auxiliary information (metadata in our case) in order to infer sensitive information. In a membership inference attack, the adversary has access to the genomic data of a victim, and tries to infer whether that victim is a part of a target genomic dataset. In attribute inference attack, the adversary tries to infer sensitive attributes using the available auxiliary information. In de-anonymization attacks, the adversary tries to link the identity of a victim with one of the participants in a genomic dataset. The most relevant attack for our scenario is membership inference attack, where the adversary (server) tries to determine from the provided metadata whether a victim is a part of one of the researcher’s dataset. In our work, the goal of each researcher is to protect the privacy of the research participants so that the server does not learn any extra information about them besides the one that was provided.

## 5 Proposed framework

Before going into the details of the proposed framework, one may refer to Table 1 which contains the frequently used notations and symbols throughout the rest of the article.

**Table 1.** Frequently used notations.

*S*	Server
$R^{i}$	Researcher *i*
$D^{s}$	Dataset used by the server to train the PCA model
$D^{i}$	Local dataset of researcher $R^{i}$
$m^{i}$	Number of SNPs in $D^{i}$
$n^{i}$	Number of individuals in $D^{i}$
*p*	Number of populations in $D^{s}$
$M^{s}$	Trained PCA model
$O^{i}$	Original PCA coordinates of researcher $R^{i}$
$C^{i}$	Noisy PCA coordinates of researcher $R^{i}$
*d*	Number of PCA dimensions
*k*	Number of clusters

Let *S* denote the server and $R^{i}$ researcher *i*. We describe the proposed framework from the researcher $R^{i}$’s point of view. The same steps are done by all participating researchers. We let $D^{i}$ represents the local dataset of researcher $R^{i}$, $m^{i}$ and $n^{i}$ represent the total number of SNPs and individuals in $D^{i}$, respectively. Before the protocol execution, the researchers establish synchronization with the server and agree on the set of SNPs to be used, as well as their order.

**Figure 1.**
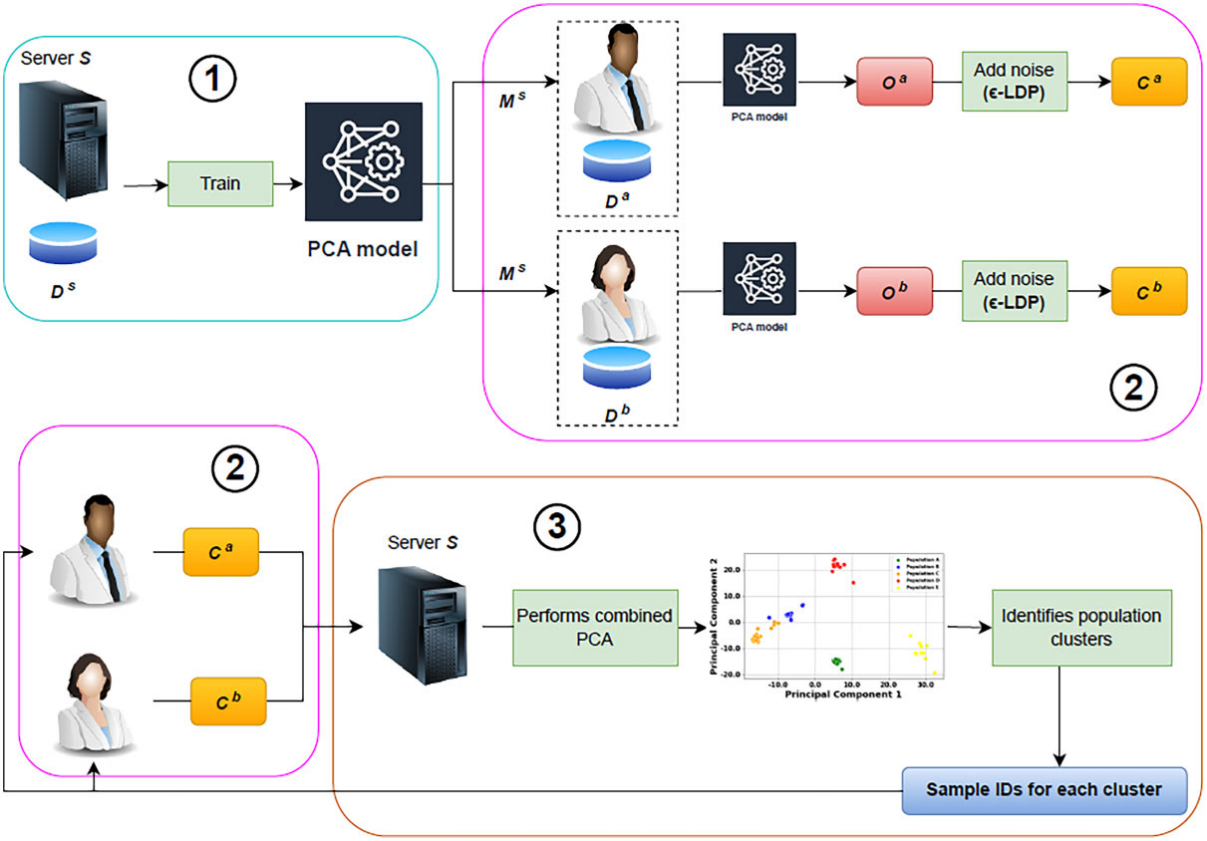
General overview of the proposed framework. In (1), the server trains the PCA model using a publicly available dataset $D^{s}$ and sends the model $M^{s}$ along with the sensitivity parameters to each researcher. In (2), each researcher uses the trained PCA model to transform their original local dataset $D^{i}$ to $O^{i}$. Next, they add Laplacian noise to $O^{i}$ in order to achieve ϵ-LDP and obtain $C^{i}$. Then, they send the metadata $C^{i}$ back to the server. In (3), the server combines the PCA results $C^{i}$ (received by the researchers) and classifies users by population clusters. Finally, the server sends the respective sample IDs along with the corresponding population cluster to each researcher and the total number of individuals that each cluster contains in the federated dataset.

*Training the PCA model*: Initially, the server *S* trains a PCA model using a publicly available genomic dataset $D^{s}$ as described in the Background Information section (step 1 in Fig. 1). We use StandardScaler from scikit-learn (Pedregosa et al. 2011) to preprocess $D^{s}$. After training the PCA model, the server sends the trained model $M^{s}$ to all researchers along with the sensitivity parameter which will be used in the noise addition step detailed below. As detailed in the Background Information, the sensitivity is calculated as the difference between the maximum and minimum values of each principal component in the public dataset $D^{s}$. Recall that $M^{s}$ includes the number of principal components (dimensions) to be used and the matrix *Q*, which contains the principal components (eigenvectors). We assume $D^{s}$ to include a wide variety of populations (*p* different populations), but not necessarily the ones in the researchers’ local datasets. In the Evaluation section, we study the impact of the *p* value (number of populations) to the accuracy of population substructure identification.

*Dimensionality reduction and noise addition at each researcher*: A researcher $R^{i}$ transforms (projects) the local dataset $D^{i}$ (which has $m^{i}$ SNPs, and hence $m^{i}$ dimensions) to *d* dimensions via the trained PCA model $M^{s}$ (step 2 in Fig. 1). In other words, the data points (i.e. the samples in the local dataset) are reduced to *d* dimensions by computing the dot product with the eigenvectors in matrix *Q*. In the rest of the discussion, we assume d=2, which can be easily extended to more dimensions. In the Evaluation section, we evaluate the performance of the proposed framework (in terms of both utility and privacy) with varying values of *d*. At the end of this step, each researcher $R^{i}$ possesses the dimensionality reduced data points of $n^{i}$ samples $O^{i}=\{O_{1}^{i},\ldots ,O_{j}^{i},\ldots ,O_{n}^{i}\}$. Here, $O_{j}^{i}=\{x_{j}^{i},y_{j}^{i}\}$, where $x_{j}^{i}$ and $y_{j}^{i}$ represent the *x* and *y* coordinates (values of principal components 1 & 2 for d=2) of sample *j’*s PCA output. In order to guarantee the privacy of the participating individuals (samples), especially against membership inference attacks, the researcher adds Laplacian noise locally to achieve ϵ-LDP. Specifically, it adds noise to the first principal component of a data point as follows: $X_{j}^{i}=x_{j}^{i}+\mathrm{Lap}\left(\frac{s_{1}}{\epsilon }\right)$, where $s_{1}$ represents the sensitivity for the first principle component. Note that the Laplace mechanism is applied separately to each dimension, and the server provides the sensitivity value for each dimension (as mentioned in the previous step). Experimental results demonstrate that the proposed approach is robust in terms of sensitivity. Specifically, varying numbers of populations in publicly available dataset $D^{s}$ causes negligible changes in the sensitivity values of each dimension. Based on Theorem 3.14 in Dwork and Roth (2013) and considering that noise addition for each dimension is independent, the privacy budget for each dimension is ϵ/2 (for d=2) and the total privacy budget is ϵ. Next, each researcher $R^{i}$ sends the metadata $C^{i}=\{C_{1}^{i},\ldots ,C_{j}^{i},\ldots ,C_{n}^{i}\}$ to the server, where $C_{j}^{i}=\{X_{j}^{i},Y_{j}^{i},ID_{j}^{i}\}$. $ID_{j}^{i}$ denotes the hashed ID of sample *j* in $D^{i}$. We hash the IDs of the research participants using SHA-256 algorithm (Penard and van Werkhoven 2008) (which is a commonly used hash function) to obfuscate their real identities from the server.

*Identification of population clusters*: After receiving the metadata from each researcher, the server generates a “combined PCA” by synthesizing the data points received from all researchers (step 3 in Fig. 1). Since all researchers use the same training model, the combined PCA generated at the server is a good approximation to the PCA obtained in a centralized setting (i.e. PCA that is obtained from the combined dataset of the researchers). As shown in the Evaluation section, we observe no utility loss when no noise is added to the data and when the dataset used by the server ($D^{s}$) to train the PCA model contains a sufficient number of populations. In other words, the trained PCA model is able to capture all the populations that are observed locally (including populations that are not part of $D^{s}$). The server uses the k-means clustering algorithm (Hartigan and Wong 1979) to determine the population cluster of each received sample ID. To determine the optimal number of clusters (populations) that are present in the combined PCA, we use the Elbow method (Syakur et al. 2018). The Elbow method works by initially plotting the explained variation in within-cluster sum of square (WCSS) as a function of the number of clusters, and then picking the number of clusters that corresponds to the elbow in the plot as the optimal number. Finally, the server sends back to the researchers (i) the population cluster label of each sample and (ii) the size of each cluster.

## 6 Privacy analysis

Providing PCA results as part of the metadata may increase the membership inference risk (i.e. inferring the existence of a target individual in a researcher’s dataset) (Homer et al. 2008; Wang et al. 2009). Here, the risk is the server inferring whether any individuals’ genome in the provided PCA output ($C^{i}$) is a match to that of a target victim considering that each data point in the PCA output corresponds to a sample in the researcher $R^{i}$’s dataset. As discussed in the Proposed Framework section, researchers use a trained PCA model by the server to generate the PCA output of the individuals in their datasets. Therefore, the server can use the same trained model to obtain the PCA output of the victim’s genome (similar to existing works, Homer et al. 2008; Sankararaman et al. 2009, we assume that the server has access to the victim’s genome). As discussed previously, we use LDP concept while sharing the PCA output. To quantify this privacy risk and identify the match (or closeness), we propose to use the Euclidian distance between the PCA outputs. Euclidian distance quantifies the length of a line segment between two points in high dimensional space, and hence it can be used to check the similarity between the PCA output of the victim’s genome and the shared PCA output of all individuals for any number of provided PCA dimensions.

In the following, we discuss our proposed power analysis (for membership inference attack) using Euclidian distance. Assume the number of dimensions in the provided PCA output is *d*. We aim to quantify the membership inference risk for a victim *v* in researcher $R^{i}$’s dataset $D^{i}$. First, we use |G|individuals from a set *G* (control set) that are not in dataset $D^{i}$. The individuals in the control group are randomly selected from the pool of all individuals that are not in $D^{i}$. For each target individual in *G*, we compute the Euclidian distance between the target *t’*s PCA output and the PCA outputs of all individuals in $D^{i}$ (considering the provided *d* dimensions) and identify the minimum Euclidian distance. Then, we identify the “Euclidian distance threshold” γ as the 5% false positive rate (for which 95% of individuals in *G* are correctly identified as not in $D^{i}$). Next, we use |*H*| target individuals from a set *H* that are in dataset $D^{i}$. For each individual in *H*, we compute the Euclidian distance between the target *v’*s PCA output and the PCA outputs of all individuals in $D^{i}$ and identify the minimum Euclidian distance. Finally, we check what fraction of these |*H*| individuals have minimum Euclidian distance that is lower than the threshold γ (i.e. correctly identified as in $D^{i}$). This fraction gives the membership inference power due to the shared PCA output. We do this analysis for different values of ϵ and *d* as discussed in the Evaluation section.

Furthermore, we also compare the privacy risk due to the shared metadata with the baseline risk of sharing GWAS statistics (which is acceptable by many institutions, e.g. NIH management of genomic summary results access 2018). We use the likelihood ratio test (LRT) to quantify the membership inference risk which is introduced due to sharing GWAS statistics (similar to previous works, Halimi et al. 2022; Sankararaman et al. 2009). As in Halimi et al. (2022), we also use the minor allele frequencies (MAF) of SNPs in order to compute the LRT. Under null hypothesis, we assume that a target individual *t* is not part of the dataset $D^{i}$ and under alternate hypothesis, we assume that the target *t* is indeed in the dataset. We formally define the overall likelihood ratio test as $\mathrm{LRT}=\sum_{s=1}^{l}z_{t,s}\;\text{log}\;\frac{a_{s}}{pop_{s}}+(1-z_{t,s})\log\frac{1-a_{s}}{1-pop_{s}},$ where $z_{t,s}$ is the value of SNP *s* of individual *t*, *l* is the total number of SNPs whose statistics are publicly released as a result of GWAS, $a_{s}$ is the MAF of SNP *s* in dataset $D^{i}$, and $pop_{s}$ is the MAF of the same SNP *s* in a reference population.

## 7 Evaluation

For evaluation, we use the openSNP dataset (Opensnp 2022) with 120 samples and 9091 SNPs and 1000 Genomes Phase 3 dataset (1000 genomes project 2021) with 600 samples and 37 751 SNPs, which were obtained after removing the SNPs with MAF values smaller than 0.01 and the ones with a missing rate greater than 5% by using PLINK (Purcell et al. 2007). Initially, we apply PCA to the openSNP dataset to detect the population substructure and later we pick a total of five different populations from the set {A, B, C, D, E} which consists of 38, 37, 24, 11, and 10 samples, respectively. We split this dataset between researchers $R^{a}$ and $R^{b}$ (to construct their local datasets), and the server (to construct the public dataset $D^{s}$).

**Figure 2.**
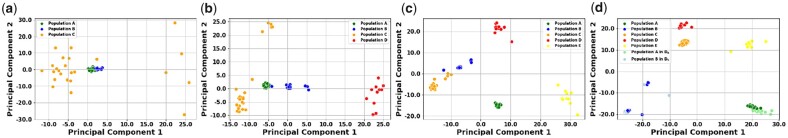
PCA plots for different number of populations in $D^{s}$. In all the scenarios, $D^{a}$ and $D^{b}$ include samples belonging to populations A and B. The populations in $D^{s}$ (used to train the PCA model) include: in (a), only population C; in (b), populations C and D; in (c), populations C, D, and E; and in (d), all five populations, also including populations A and B, which are shown in lighter colors.

Initially, we evaluate the impact of using a different number of populations at the server’s dataset $D^{s}$ which is used to train the PCA model. We let $D^{a}$ and $D^{b}$ include 20 and 18 samples, respectively, and each of them has a mix of samples belonging to populations A and B. The rest of the samples are used as part of $D^{s}$ depending on the experiment. We design four experiments by changing the content (number of unique populations) of the server’s public dataset $D^{s}$. We first run the PCA in a centralized setting (i.e. by combining all the datasets $D^{a}$, $D^{b}$, $D^{s}$) and cluster the samples (by population) using k-means clustering algorithm on the PCA output. We mark the obtained clusters as ground truth (i.e. all the samples are correctly classified to the corresponding population) and use them as a benchmark. Then, we evaluate the proposed framework by changing the content of $D^{s}$ and compute the accuracy of the proposed technique with respect to the benchmark. We show the results in Fig. 2. We observe an accuracy of 0.92, 0.97, and 1 when $D^{s}$ contains samples from population {C}, populations {C, D}, and populations{C, D, E}, respectively (as shown in Fig. 2a–c). In Fig. 2d, the server trains the PCA model on all five populations, also including A and B, and our proposed technique achieves an accuracy of 1. Note that the samples from populations A and B in $D^{s}$ which are used to train the PCA model are different from the ones in $D^{a}$ and $D^{b}$. Overall, we observe that (i) the performance (accuracy) of our framework increases as the number of different populations increases in $D^{s}$, (ii) the performance (accuracy) of our framework achieves the benchmark accuracy when $D^{s}$ includes more than two different populations, and (iii) even when the trained PCA model on $D^{s}$ does not fully represent all locally observed populations, we still achieve high accuracy. We obtain similar results for different combinations of the five populations in $D^{a}$, $D^{b}$, and $D^{s}$.

Next, we evaluate the trade-off between utility and privacy of our proposed framework on 1000 Genomes dataset (which has a bigger size and contains three different populations). We use 150 samples to train the PCA model and the rest (450 samples) as researchers’ data. We denote the total number of samples in all researchers’ data as $n^{t}$. We pick precision and recall as utility metrics, as there are no true-negatives in the results. As discussed previously in the Proposed Framework section, the server aims to correctly classify each sample to the corresponding population cluster based on the metadata sent by the researcher. Here, precision is the ratio of the number of correctly classified individuals to a cluster over the total number of classified individuals to the same cluster. The other utility metric is recall or the true positive rate, which is the ratio of the number of correctly classified individuals to a cluster over the total number of the individuals belonging to that cluster. We use power analysis to measure the privacy risk due to membership inference attack, as defined in the Privacy Analysis section. A lower value of power implies a lower risk for the membership inference attack.

For each experiment, we create the local datasets $D^{a}$ and $D^{b}$ belonging to researchers $R^{a}$ and $R^{b}$, by randomly selecting samples from two populations out of three. On the other hand, we use samples from all three populations as part of $D^{s}$. We measure the precision, recall, and power for different values of ϵ (from 0.1 to 5 in steps of 0.1). We repeat each experiment 100 times and report the average of the results. Additionally, we also vary the initial setting of the k-means clustering algorithm, which is the number of clusters (denoted as *k*) the model is capable to predict in order to examine the impact of *k* on the utility and power metrics. In our case, the minimum value of clusters is 2 and the optimum is 3 among all ϵ values, which is computed by the Elbow method and is also equal to the total number of populations available in the dataset.

**Figure 3.**
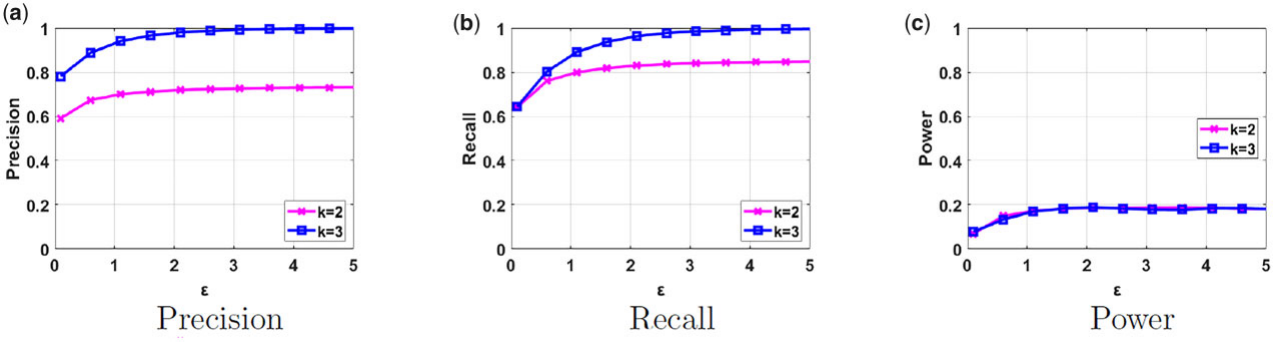
Precision, recall, and power results of our proposed framework in parts (a), (b), and (c), respectively, for different values of ϵ and *k* when both $D^{a}$ and $D^{b}$ contain samples from one population each.

We first consider the scenario that $D^{a}$ and $D^{b}$ contain only one type of population each (different from each other). The results are shown in Fig. 3. As ϵ increases (i.e. the amount of noise added decreases), our framework achieves higher utility values, but at the same time, we observe higher power values for membership inference as well. For ϵ=3 and k=3, we observe a precision and recall of almost 1, and a power of 0.2. As the number of clusters (*k*) in the k-means clustering algorithm increases from 2 to 3, utility in terms of both precision and recall increases. This shows that the proposed framework has better performance when the selected number of clusters is close to the optimum. Note that the power keeps increasing for larger ϵ values (Supplementary Fig. S1 illustrates this) and the power reaches 1 for ϵ=∞.

**Figure 4.**
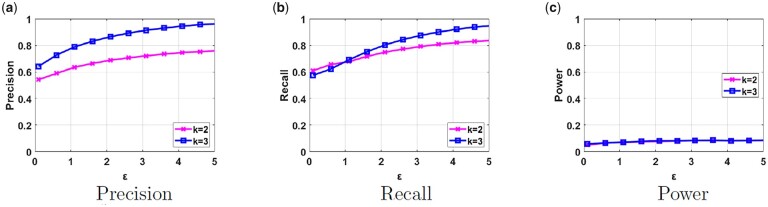
Precision, recall, and power results of our proposed framework in parts (a), (b), and (c), respectively, for different values of ϵ and *k* when both $D^{a}$ and $D^{b}$ contain samples from two different populations.

We also consider a more complex scenario where both datasets $D^{a}$ and $D^{b}$ contain samples from the same two populations (the individuals in $D^{a}$ and $D^{b}$ do not overlap). At the same time, this is a more realistic assumption since a shared dataset may contain multiple populations. In Fig. 4, we observe similar results as in Fig. 3 in terms of utility (slightly lower utility for k=3). We still notice gradually increasing trends in precision and recall when ϵ increases. We also observe that the membership inference power is halved compared to the one obtained in the previous scenario. Even though the attacker can determine the population of a target victim, it is hard for it to determine to which dataset it belongs due to the fact that both researchers’ datasets contain samples from the same two populations.

**Figure 5.**
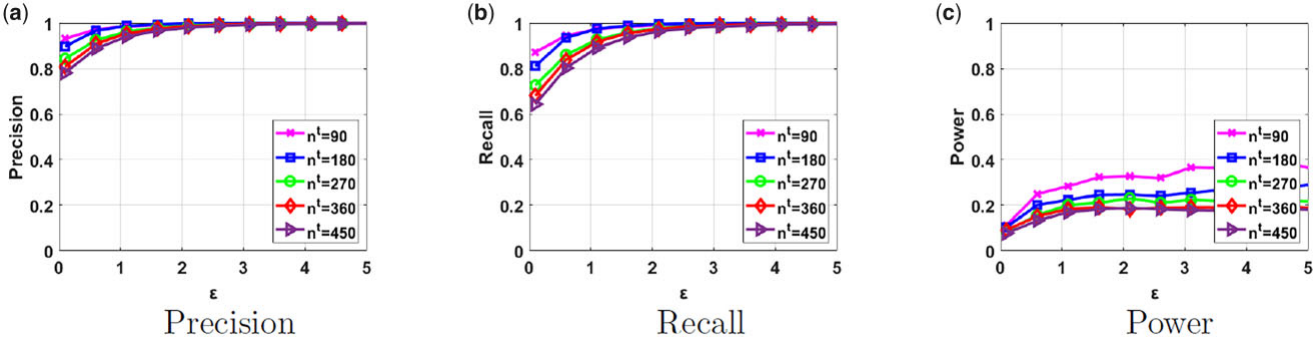
Precision, recall, and power results of our proposed framework in parts (a), (b), and (c), respectively, for different number of samples in the researchers’ datasets $n^{t}$. $D^{a}$ and $D^{b}$ contain samples from one population each and k=3.

Furthermore, we also evaluate the impact of the total number of samples $n^{t}$ that are part of the researchers’ datasets. We present the results in Fig. 5 for k=3 when $D^{a}$ and $D^{b}$ contain samples from one type of population each. We observe that for higher values of $n^{t}$, we obtain slightly lower values for both utility metrics and the power of membership inference attack. Next, we evaluate the effect of providing different number of principal components *d*, which also corresponds to the number of dimensions of the PCA output (Supplementary Fig. S2). We noticed that the results for different number of dimensions were very similar in terms of both utility and membership inference power. For most of the ϵ values, the utility and power values were almost identical. Considering that usage of a higher *d* would introduce computation overhead, we only consider the first two principal components (d=2).

The main factors that affect the running time of our proposed framework at the server are the sample size of the local datasets $n^{t}$ and the population heterogeneity at the researchers’ datasets. We conduct a few experiments for different number of populations (1 or >1) and different values of $n^{t}$ and inspect the change in the running time (see Supplementary Table S1 for the numerical results). We observe a linear increase in the running time as the number of samples per dataset increases. We also notice that the running time on the researcher side doubles when the samples in each researcher’s dataset belong to multiple populations. Considering that the running time of the framework for a dataset with over 37 000 SNPs and 600 samples is <1 s, we conclude that the proposed framework is practical and efficient.

Finally, we compare the privacy risk of our proposed framework due to sharing the PCA output with the one due to sharing GWAS statistics (which we refer to as the “baseline risk” that is accepted by many institutions, including the NIH, NIH management of genomic summary results access 2018). As previously discussed in the Privacy Analysis section, we use LRT to quantify the power of membership inference attack due to sharing GWAS statistics for different number of SNPs. We obtain a power >0.44 when as few as 10 statistics (MAF values of SNPs) are provided as a result of GWAS on a dataset containing 120 samples and this power increases as the number of returned statistics increases. Although there is no standard number of statistics that are released as a result of GWAS, typically researchers may share tens of statistics as the research outcome. If we make a cross comparison with the power achieved in Figs. 3–5, we notice that the power of the Euclidian distance on the PCA output does not exceed 0.4. Note that the line for $n^{t}=90$ (the pink line) in Fig. 5c contains a similar number of samples in the dataset with the above experiment, and still produces fairly low power for different ϵ values. Hence, we conclude that the privacy risk of the proposed scheme is lower than the risk posed due to sharing of GWAS statistics.

## 8 Conclusion

In this work, we have proposed a novel and effective privacy-preserving framework which partitions populations in collaborative studies. Via experiments, we have shown that the proposed framework identifies with high accuracy, precision, and recall the genetic differences among collaborators’ datasets while preserving the privacy of the research participants. We have empirically shown that our proposed framework keeps the privacy risk below the baseline risk of sharing summary statistics. This work will enable researchers to conduct collaborative research with high quality data while ensuring that the privacy of the research participants is preserved.

## Supplementary Material

btad274_Supplementary_Data

## Data Availability

The source code of the proposed framework and above experiments can be found at https://github.com/wxl387/Privacy-Preserving-Population-Stratification-for-Collaborative-Genomic-Research.
