# Supplementary figures and images for: Privacy preserving identification of population stratification for collaborative genomic research
Source: Bioinformatics. 2023 Jun 30;39(Suppl 1):i168–76. doi: 10.1093/bioinformatics/btad274 (PMC10311306; doi:10.1093/bioinformatics/btad274)

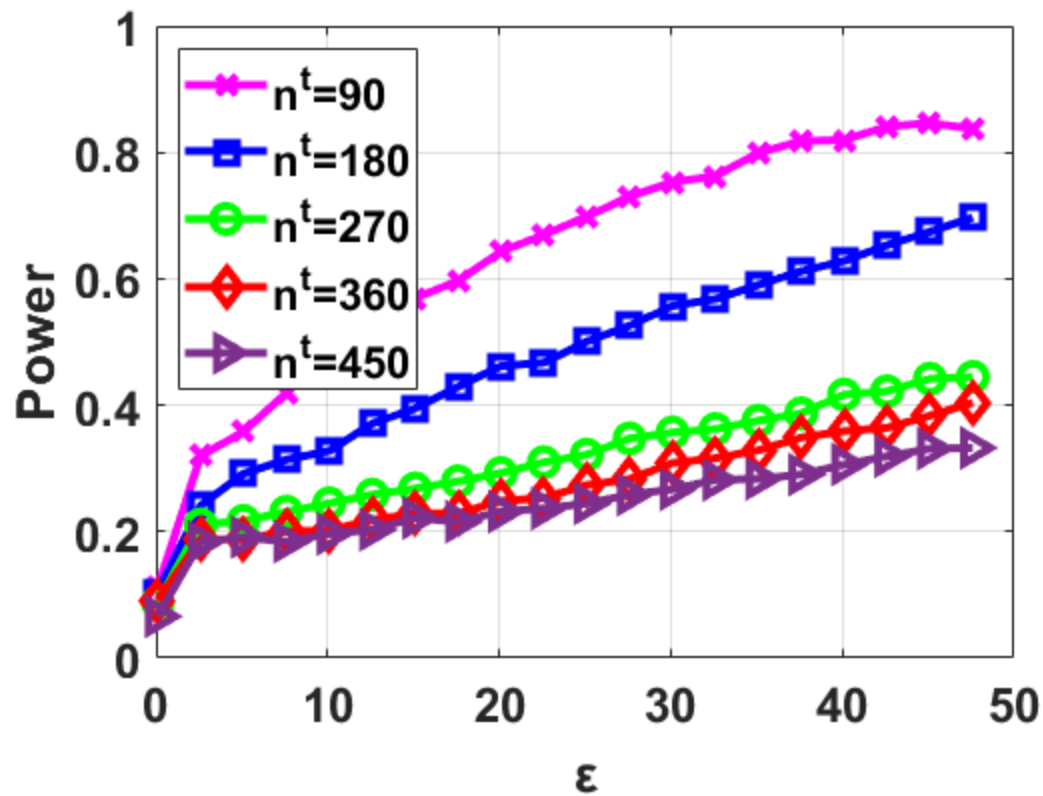

Supplement: btad274_Supplementary_Data [file btad274_supplementary_data.zip › btad274_Supplementary_Data/Dervishi.234.fig.1.pdf]

Precision

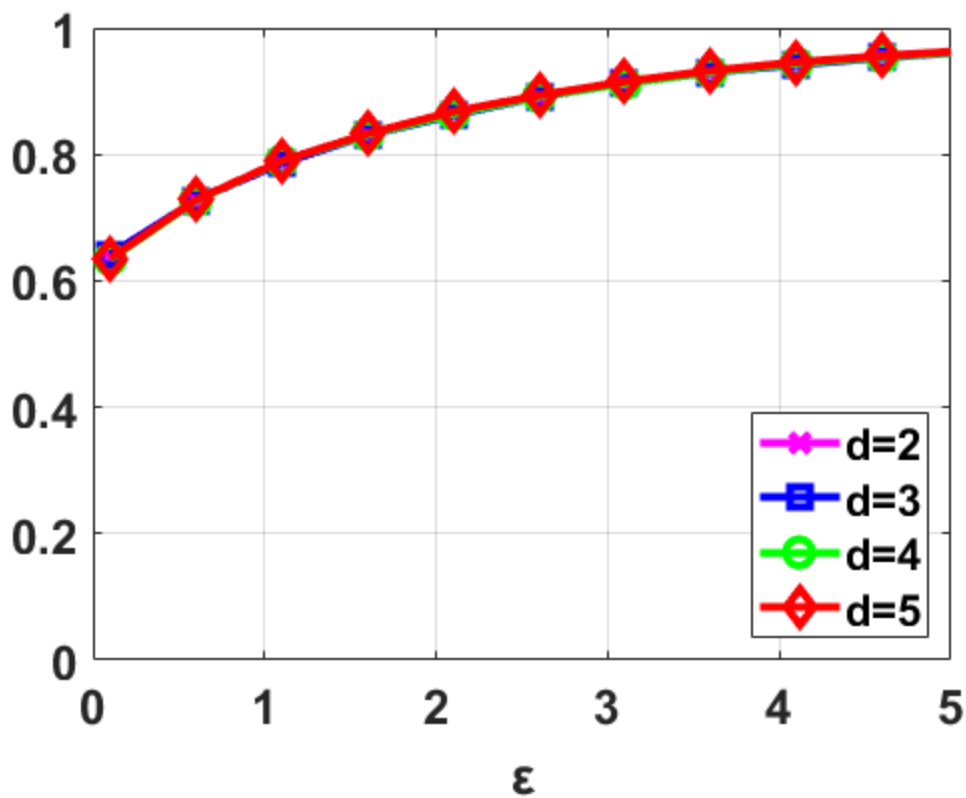

Supplement: btad274_Supplementary_Data [file btad274_supplementary_data.zip › btad274_Supplementary_Data/Dervishi.234.fig.2.a.pdf]

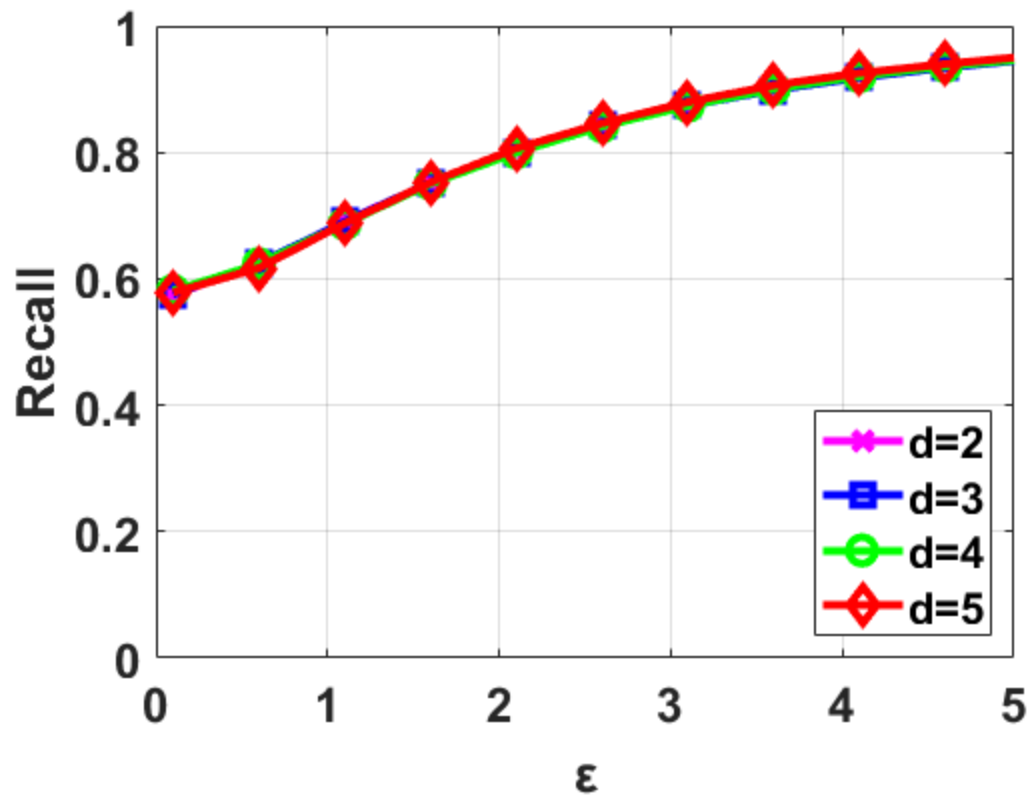

Supplement: btad274_Supplementary_Data [file btad274_supplementary_data.zip › btad274_Supplementary_Data/Dervishi.234.fig.2.b.pdf]

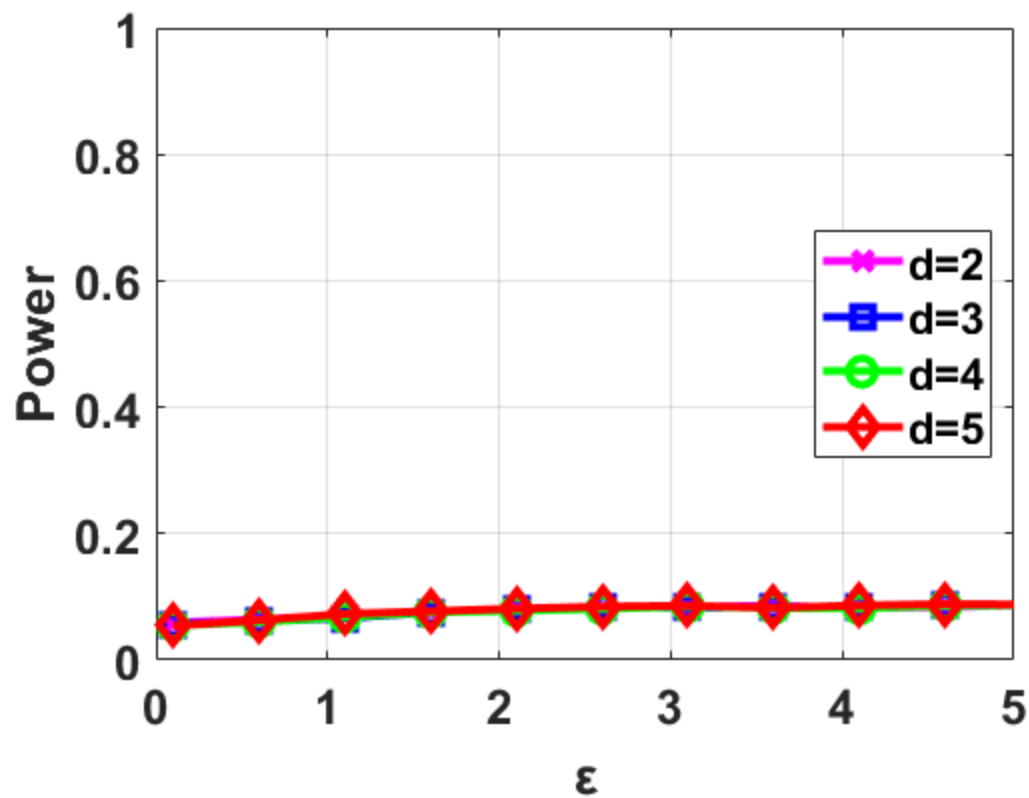

Supplement: btad274_Supplementary_Data [file btad274_supplementary_data.zip › btad274_Supplementary_Data/Dervishi.234.fig.2.c.pdf]

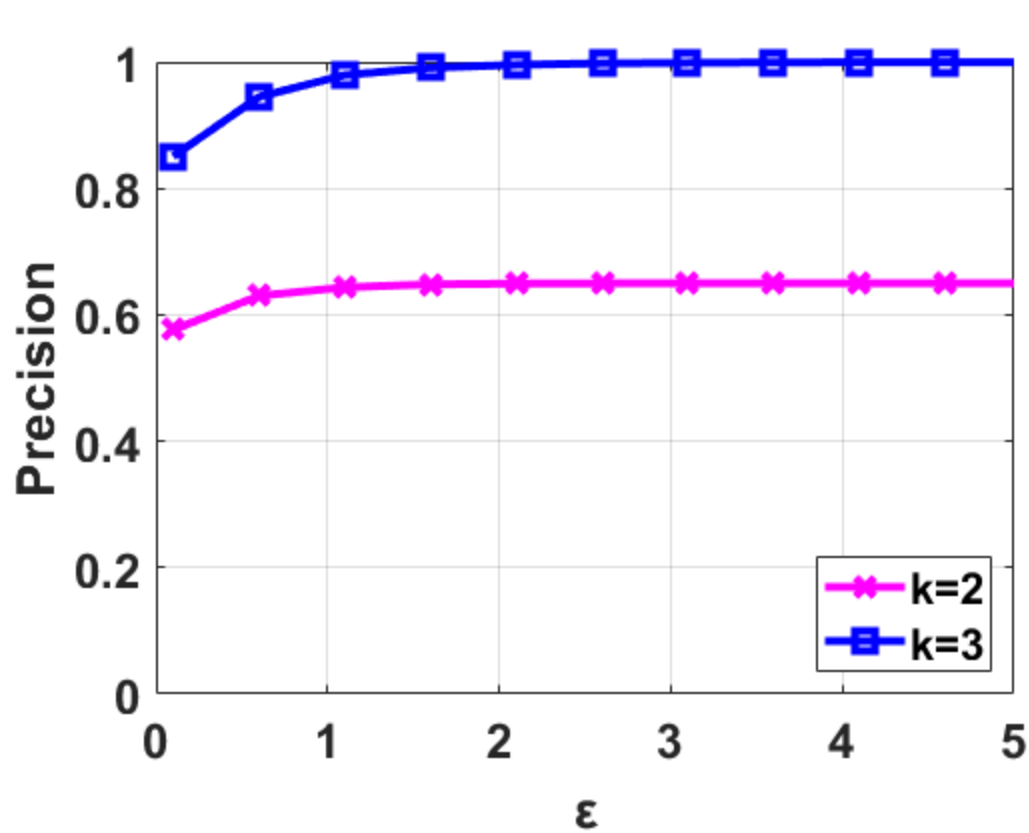

Supplement: btad274_Supplementary_Data [file btad274_supplementary_data.zip › btad274_Supplementary_Data/Dervishi.234.fig.3.a.pdf]

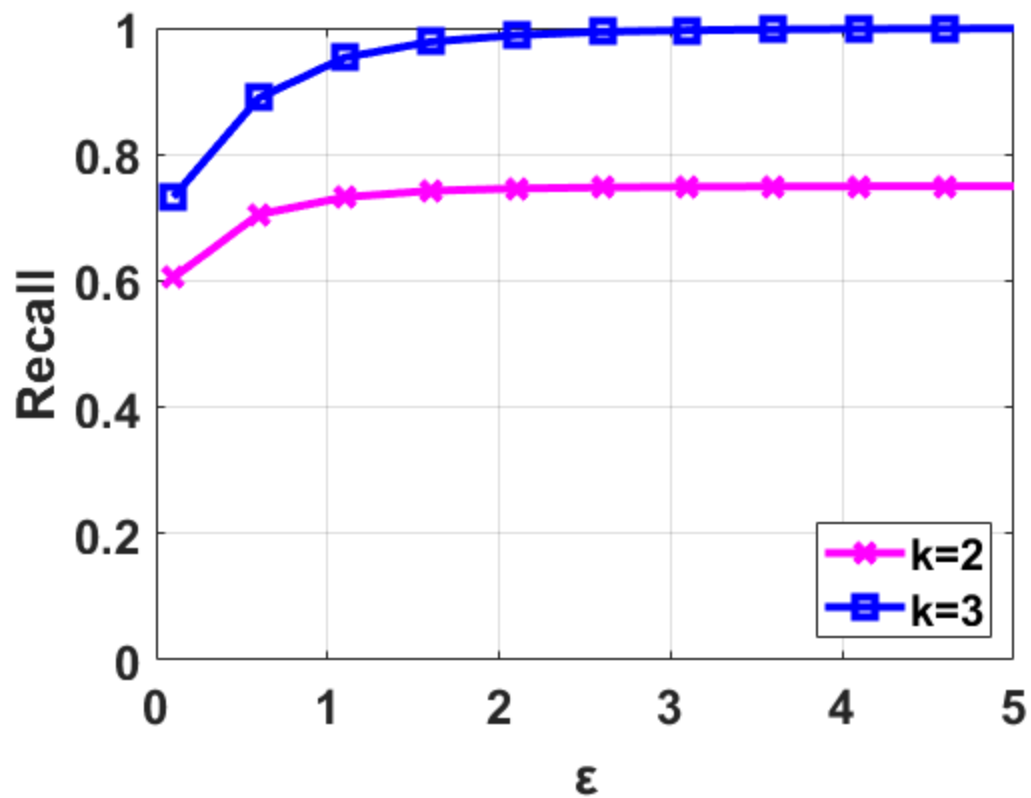

Supplement: btad274_Supplementary_Data [file btad274_supplementary_data.zip › btad274_Supplementary_Data/Dervishi.234.fig.3.b.pdf]

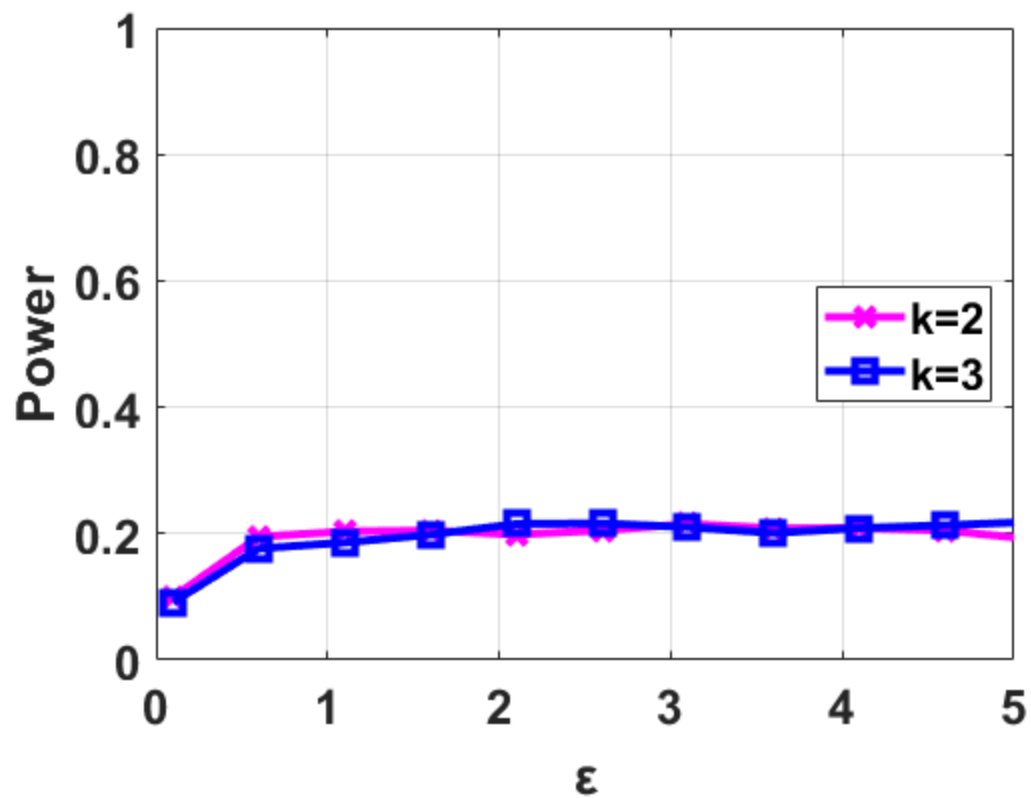

Supplement: btad274_Supplementary_Data [file btad274_supplementary_data.zip › btad274_Supplementary_Data/Dervishi.234.fig.3.c.pdf]

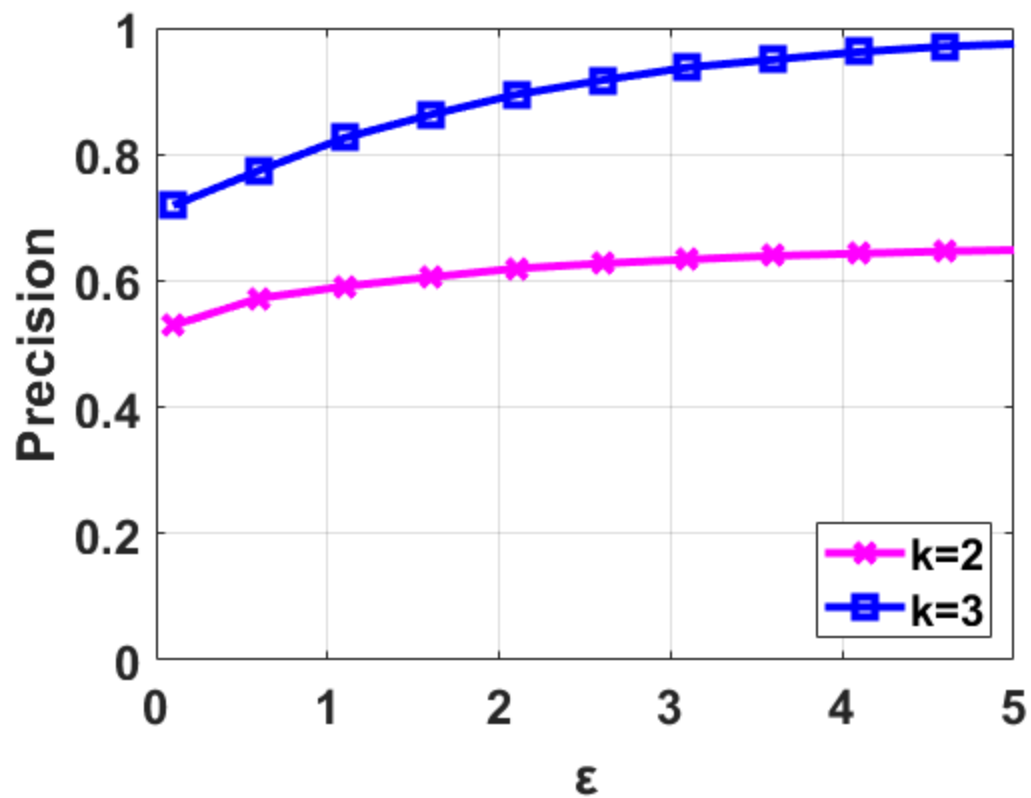

Supplement: btad274_Supplementary_Data [file btad274_supplementary_data.zip › btad274_Supplementary_Data/Dervishi.234.fig.4.a.pdf]

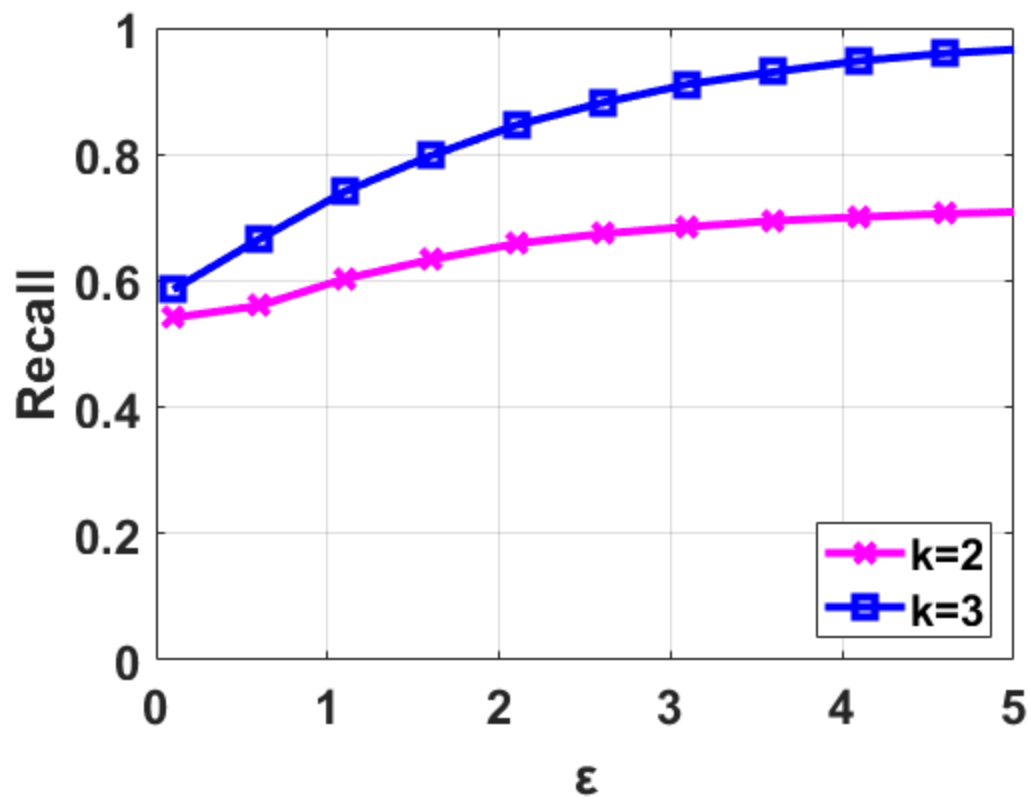

Supplement: btad274_Supplementary_Data [file btad274_supplementary_data.zip › btad274_Supplementary_Data/Dervishi.234.fig.4.b.pdf]

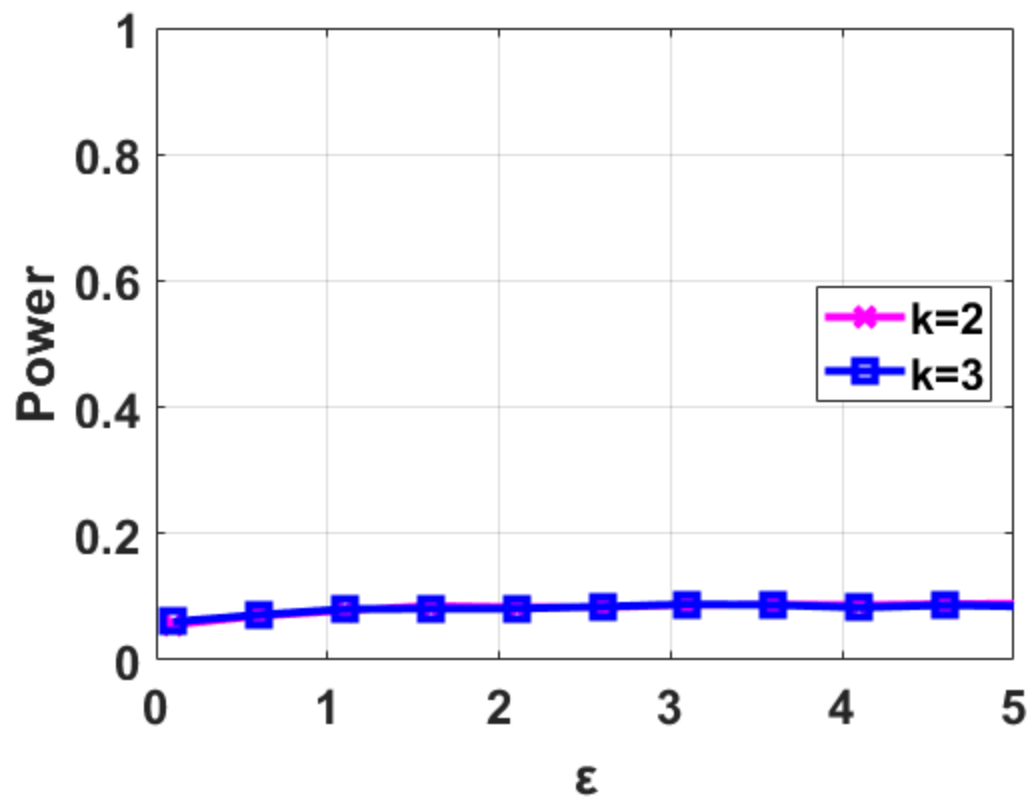

Supplement: btad274_Supplementary_Data [file btad274_supplementary_data.zip › btad274_Supplementary_Data/Dervishi.234.fig.4.c.pdf]
